# Cathelicidin attenuates hyperoxia-induced kidney injury in newborn rats

**DOI:** 10.1080/0886022X.2019.1651741

**Published:** 2019-08-19

**Authors:** Hsiu-Chu Chou, Chung-Ming Chen

**Affiliations:** a Department of Anatomy and Cellular Biology, School of Medicine, College of Medicine, Taipei Medical University, Taipei, Taiwan;; b Department of Pediatrics, School of Medicine, College of Medicine, Taipei Medical University, Taipei, Taiwan

**Keywords:** Antimicrobial peptide, collagen, inducible nitric oxide synthase, nuclear factor-κB, oxidative stress, Ym-1

## Abstract

**Aim:** Supplemental oxygen is often used to treat neonates with respiratory disorders. Human and animal studies have demonstrated that neonatal hyperoxia increases oxidative stress and induces damage and collagen deposition in kidney during the perinatal period. Cathelicidin LL-37 is one important group of human antimicrobial peptides which exhibits antioxidant activity and its overexpression resists hyperoxia-induced oxidative stress. This study was designed to evaluate the protective effects of cathelicidin in hyperoxia-induced kidney injury in newborn rats.

**Methods:** Sprague-Dawley rat pups were reared in either room air (RA) or hyperoxia (85% O_2_) and were randomly treated with low-dose (4 mg/kg) and high-dose (8 mg/kg) cathelicidin in normal saline (NS) administered intraperitoneally on postnatal days 1–6. The following six groups were obtained: RA + NS, RA + low-dose cathelicidin, RA + high-dose cathelicidin, O_2_ + NS, O_2_ + low-dose cathelicidin, and O_2_ + high-dose cathelicidin. Kidneys were taken for Western blot and histological analyses on postnatal day 7.

**Results:** The hyperoxia-reared rats exhibited significantly lower body weights and anti-inflammatory M2 macrophages, but the kidney injury scores, oxidative stress marker 8-hydroxy-2'-deoxyguanosine (8-OHdG)-positive cells, pro-inflammatory M1 macrophages, collagen deposition, and NF-κB expression were higher than did the RA-reared rats.

**Conclusions:** Cathelicidin treatment attenuated kidney injury as evidenced by lower kidney injury scores, 8-OHdG-positive cells, collagen deposition, and reversion of hyperoxia-induced M1/M2 macrophage polarization. The role of Cathelicidin in ameliorates kidney injury of the hyperoxia newborn rats was accompanied by decreased NF-κB expression, which probably through the modulating NF-κB activity in the kidney.

## Introduction

Supplemental oxygen is often used to treat newborns with respiratory disorders. However, oxygen therapy provided to infants has adverse effects. Human and animal studies have demonstrated that neonatal hyperoxia increases oxidative stress and induces glomerular and tubular damage. These are manifested as enlarged renal corpuscles, renal tubular necrosis, interstitial inflammation, and kidney fibrosis during the perinatal period [1–4].

Newborn infants are often needed to be resuscitated by high concentration of oxygen. These result in accumulation oxidative stress of the newborn [5,6]. Oxidative stress has been implicated in a number of common diseases of prematurity including retinopathy of prematurity, bronchopulmonary dysplasia, and necrotizing enterocolitis [7,8]. Cathelicidins are a family of antimicrobial peptides with anti-bacterial, anti-viral, and anti-fungal properties [9]. Cathelicidin LL-37 is the sole cathelicidin has been characterized in humans; this peptide is derived by proteolysis from the C-terminal end of the human CAP18 protein with 37 amino acids [10]. It had been known that LL-37 is induced by stimuli of inflammation or infections [11] and has antimicrobial ability against both Gram-positive and Gram-negative bacteria [12]. In addition to its antimicrobial activity, LL-37 has potent chemotactic and immunomodulatory properties [13].

Antimicrobial peptides serving as natural antibiotics, widely distributed across the organism, mainly in mucus layer [14]. Innate immunity and the antimicrobial peptide cathelicidin appear to play important roles in the pathogenesis of both urinary tract infection and diarrhea-associated hemolytic uremic syndrome [15]. Overexpression of antimicrobial peptide has been shown to resist hyperoxia-induced oxidative stress by increasing antioxidant enzyme activities and preventing an increase in reactive oxygen species levels in *Drosophila melanogaster* flies [16]. Cao et al. [17] identified cathelicidin from amphibian exhibits antioxidant activity. Cathelicidin LL-37 significantly inhibited the expression of specific pro-inflammatory genes upregulated by nuclear factor-κB (NF-κB) in the presence of lipopolysaccharide [18]. In this study, we hypothesized that hyperoxia exposure would increase kidney oxidative stress and NF-κB activity and cathelicidin would ameliorate kidney injury through inhibition of oxidative stress and NF-κB activity in newborn rats.

## Materials and methods

### Animals

This study was performed in accordance with guidelines provided by the Animal Care Use Committee of Taipei Medical University and was approved by the committee. Time-dated pregnant Sprague-Dawley rats were housed in individual cages with free access to laboratory food and water maintained on a 12:12-h light–dark cycle and was allowed to deliver vaginally at term. Within 12 h of birth, the litters were pooled and randomly redistributed among the newly delivered mothers, and the pups were then randomly assigned to receive either room air (RA) or O_2_-enriched atmosphere treatment. The pups in the O_2_-treated (normobaric hyperoxia) group were reared in an atmosphere containing 85% O_2_ from postnatal days 1–7. The pups in the RA group were reared in RA from postnatal days 1–7. The kidneys used for these experiments were obtained from a previous study designed to assess intestinal injury [19].

Half of the pups from the RA and hyperoxia exposure were treated with low-dose and high-dose cathelicidin (Human LL-37 (LLGDFFRKSKEKIGKEFKRIVQRIKDFLRNLVPRTES) (4 and 8 mg/kg, respectively, Bioworld Technology, Inc., St Louis Park, MN) in 0.05 mL normal saline (NS) were administered intraperitoneally from postnatal days 1–6. The dosage was based and modified from Song et al. [20]. Six study groups were obtained as follows: (1) RA + NS; (2) RA + low-dose cathelicidin; (3) RA + high-dose cathelicidin; (4) O_2_ + NS; (5) O_2_ + low-dose cathelicidin; and (6) O_2_ + high-dose cathelicidin. To avoid O_2_ toxicity in the nursing mothers, they were rotated between the O_2_ treatment and RA control litters every 24 h. The O_2_-rich atmosphere was maintained in a transparent 40 × 50 × 60-cm plexiglass chamber receiving O_2_ continually at 4 L/min. O_2_ levels were monitored using a ProOx P110 monitor (NexBiOxy, Hsinchu, Taiwan). Body weights were recorded and the kidneys were harvested on postnatal day 7.

### Histology

The kidney was placed in 4% paraformaldehyde, washed in phosphate-buffered saline, and serially dehydrated in increasing concentrations of ethanol prior to being embedded in paraffin. Five-micrometer tissue sections were stained with hematoxylin and eosin and Masson’s trichrome; examined using light microscopy; and subsequently assessed for kidney morphology and fibrosis. The histological analysis of the kidney was modified according to the suggestions of Toledo-Rodriguez et al. [21]. The fraction of the cortex occupied by glomeruli was calculated as the ratio of the grid points that touched the cortex to the grid points that touched glomeruli. The sizes of the individual glomeruli located in the middle cortex and juxtamedullary zone were calculated as the average of the largest and smallest glomerular diameters within a field of view; the calculations involved 10 ± 5 glomeruli per kidney defined tubular injury as tubular dilation, tubular atrophy, vacuolization, the degeneration and sloughing of tubular epithelial cells, or thickening of the tubular basement membrane [22]. Only cortical tubules were used in the scoring system, where 0 = no tubular injury; 1= <10% of tubules injured; 2 = 10–25% of tubules injured; 3 = 26–50% of tubules injured; 4 = 51–75% of tubules injured; and 5= >75% of tubules injured.

### Immunohistochemistry for 8-OHdG, NF-κB, iNOS, and YM-1

Immunohistochemical staining was performed on 5-μm paraffin sections using immunoperoxidase visualization. After routine deparaffinization, heat-induced epitope retrieval was performed by immersing the slides in 0.01 M sodium citrate buffer (pH 6.0). To block the endogenous peroxidase activity and nonspecific binding of antibodies, the sections were preincubated for 1 h at room temperature in 0.1 M phosphate-buffered saline containing 10% normal goat serum and 0.3% H_2_O_2_. The sections were then incubated for 20 h at 4 °C with mouse monoclonal anti-8-hydroxy-2'-deoxyguanosine (8-OHdG) antibody (1:100; Abcam Inc., Cambridge, MA), anti-NF-κB (1:100; Thermo Scientific, Rckford, IL), rabbit polyclonal anti-inducible nitric oxide synthase (iNOS) (1:100; Thermo Fisher Scientific, Rockford, IL), or rabbit polyclonal anti-Ym-1 (1:25; STEMCELL Technologies Inc., Vancouver, Canada) as primary antibodies. The sections were then treated for 1 h at 37 °C with biotinylated goat anti-mouse or rabbit IgG (1:200, Jackson ImmunoResearch Laboratories Inc., West Grove, PA, USA). Following the reaction produced using reagents from an ABC kit (Avidin-Biotin Complex, Vector Laboratories, Inc., Burlingame, CA), the reaction products were visualized using a diaminobenzidine substrate kit (Vector Laboratories, Inc.) according to the recommendations of the manufacturer. 8-OHdG staining was quantified by considering the positively stained nuclei per high-power field. Positively stained cells were counted in five fields randomly selected from each section using a light microscope at ×400 magnification and results were expressed as the percentage of positively stained nuclei to total cells. Positive 8-OHdG cell nuclei were scored in five randomly selected fields from each section at 400× magnification were captured using a digital camera and imported into a computerized image analysis system (Image-Pro Plus version 5.1 for Windows, Media Cybernetics, Silver Spring, MD, USA). An automatic object counting and measuring process was used to quantify the immunoreactivity-positive 8-OHdG cell nuclei. This generated a percentage of positively stained cells and values were expressed as a labeling index (%). The optical density values of NF-κB, iNOS, and YM-1 stained renal sections were evaluated evaluate in five non-overlapping microscopic fields per animal using Image Pro Plus version 6.0 (Media Cybernetics; Bethesda, MD) [23].

### Statistical analysis

Data are presented as means ± SD. Analysis of difference was determined using two-way ANOVA and analysis of significance was based on Bonferroni’s correction for multiple comparisons. Differences were considered significant at *p*<.05.

## Results

Six dams gave birth to a total of 60 pups. The litters were pooled and ten pups were randomly redistributed to the newly delivered mothers. Three litters each were assigned to RA and hyperoxia groups, respectively. Ten rats reared in RA and treated with NS or cathelicidin and ten rats reared in hyperoxia and treated with cathelicidin all survived. One rat pup reared in hyperoxia and treated with NS died on postnatal day 6.

### Body weight at birth and on postnatal day 7

The body weights of the pups assigned to RA (7.26 ± 0.76, 7.23 ± 0.77, and 7.28 ± 0.30 g) or O_2_-enriched air (7.28 ± 0.48, 7.52 ± 0.55, and 7.25 ± 0.63 g) and treated with NS, low-dose cathelicidin, or high-dose cathelicidin were comparable at birth. The body weights of the rats in the hyperoxia groups were significantly lower than those of the rats in the RA groups (13.51 ± 1.19, 13.81 ± 1.57, and 13.64 ± 0.60 g) on postnatal day 7. The rats reared in hyperoxia and treated with high-dose cathelicidin (11.92 ± 0.83 g) exhibited a significantly higher body weight compared with the rats reared in hyperoxia and treated with NS or low-dose cathelicidin (10.38 ± 0.73 and 10.23 ± 0.53 g).

### Histology

**Figure 1.**
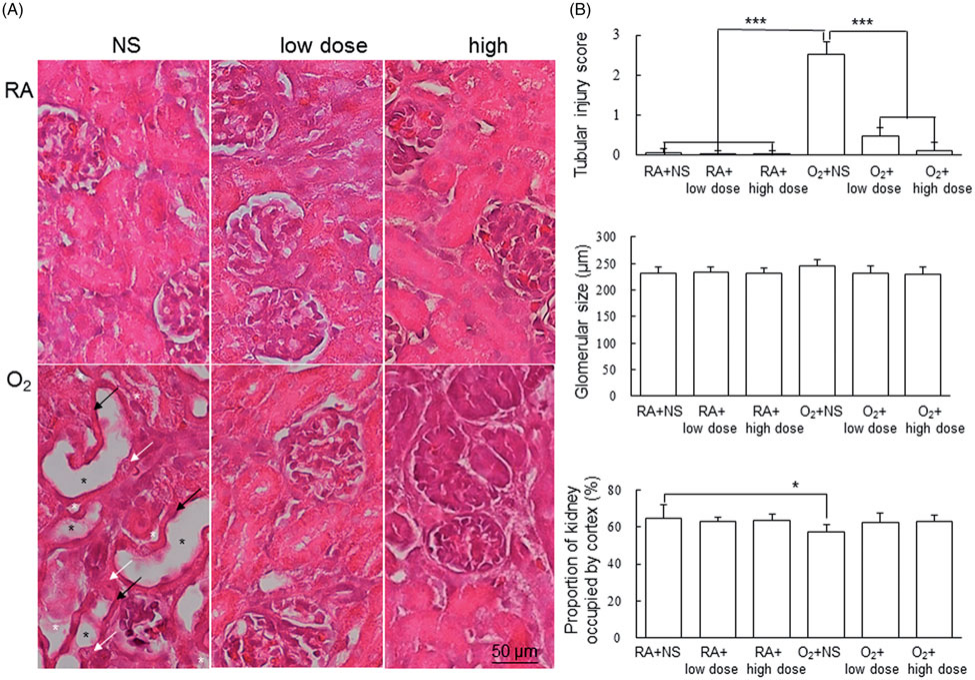
Histology and kidney injury score on postnatal days 7. (A) Representative photomicrographs of kidney and (B) tubular injury score, glomerular size, and proportion of kidney occupied by cortex in rats reared in RA or O_2_-enriched air and treated with NS, low-dose cathelicidin, or high-dose cathelicidin. The rats reared in RA and treated with NS or cathelicidin exhibited normal kidney framework. The rats reared in hyperoxia and treated with NS exhibited tubular atrophy (black arrow), dilatation of the tubular lumen (black asterisk), vacuolar degeneration of the tubular epithelia (white arrow), and increased space between the renal tubules (white asterisk). The rats reared in O_2_-enriched air exhibited a significantly higher tubular injury score and lower proportion of kidney occupied by cortex than did the rats reared in room air and cathelicidin treatment significantly decreased hyperoxia-induced increase in tubular injury score. Data are shown as the mean ± SD. *p<.05, ***p<.001. NS: normal saline; RA: room air.

Figure 1(A) shows representative kidney sections stained with hematoxylin and eosin from the RA-reared and hyperoxia-reared rats and treated with NS or cathelicidin on postnatal day 7. Tubular atrophy, dilatation of the tubular lumen, vacuolar degeneration of the tubular epithelia, and increased space between the renal tubules were observed in the hyperoxia-reared rats. The RA-reared rats treated with NS or cathelicidin exhibited no tubular injuries. The hyperoxia-reared rats exhibited significantly higher tubular injury scores than did the RA-reared rats treated with NS or cathelicidin, and cathelicidin treatment significantly reduced the hyperoxia-induced increase in tubular injury scores (Figure 1(B)). Hyperoxia exposure and cathelicidin treatment demonstrated similar effects on glomerular size though the differences were not statistically significant (Figure 1(B)).

### Hyperoxia effect on renal oxidative stress

**Figure 2.**
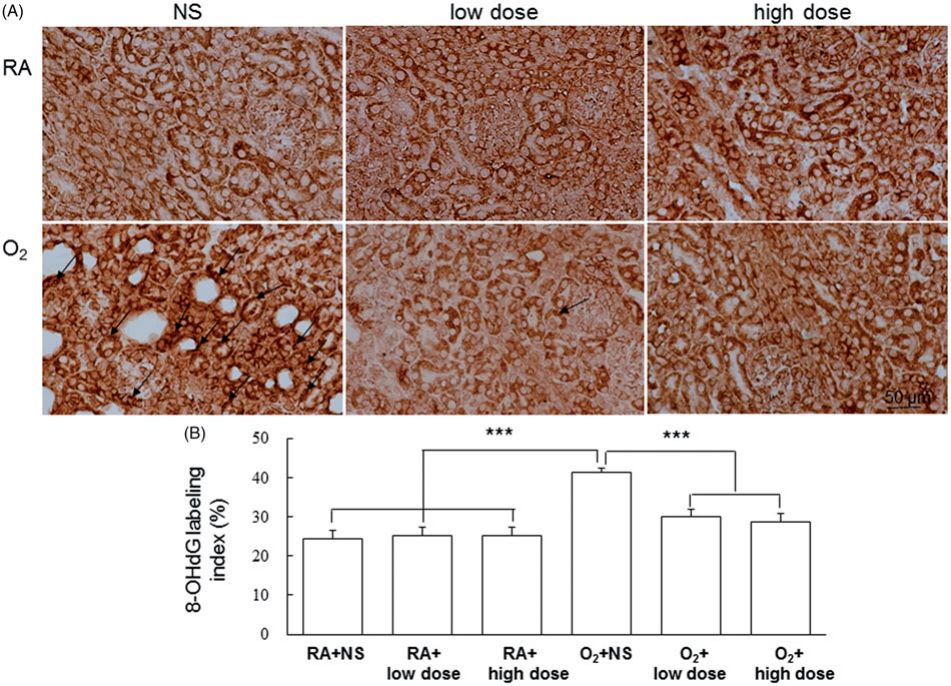
(A) Representative immunohistochemistry of 8-hydroxy-2'-deoxyguanosine (8-OHdG) and (B) quantitative analysis of 8-OHdG-positive cells on postnatal days 7. The rats reared in O_2_-enriched air and treated with NS exhibited significantly more 8-OHdG-positive cells (arrow) than did the RA-reared rats treated with NS or cathelicidin, and cathelicidin treatment reduced the hyperoxia-induced increase in 8-OHdG-positive cells. Data are shown as the mean ± SD. ***p<.001. NS: normal saline; RA: room air.

The oxidative stress marker 8-OHdG was positively stained at the glomerular and tubular cell nuclei (Figure 2(A)). The hyperoxia-reared rats treated with NS exhibited significantly more 8-OHdG-positive cells than did the RA-reared rats treated with NS or cathelicidin, and cathelicidin treatment reduced the hyperoxia-induced increase in 8-OHdG-positive cells (Figure 2(B)).

### Immunohistochemistry of NF-κB

**Figure 3.**
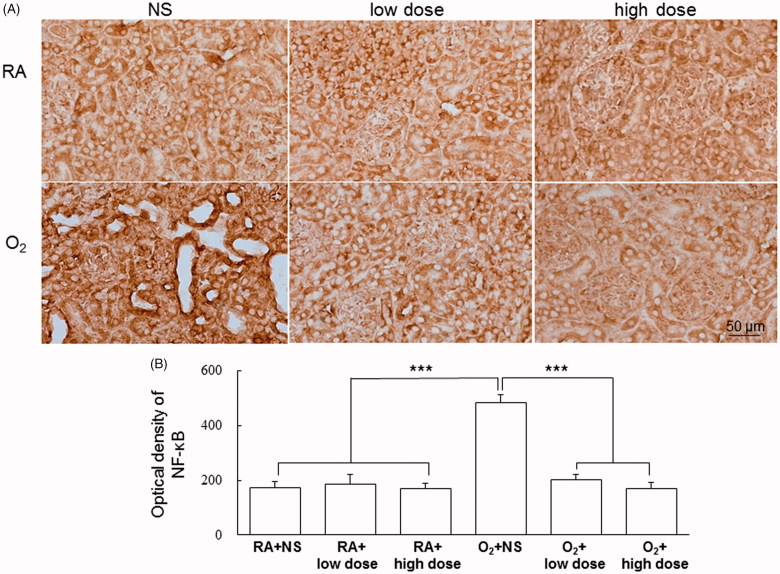
(A) Representative immunohistochemistry and (B) optical density of NF-κB in the RA-reared and hyperoxia-reared rats and treated with NS or cathelicidin on postnatal day 7. The hyperoxia-reared rats exhibited more NF-κB intensity than did the RA-reared rats treated with NS or cathelicidin. Cathelicidin treatment reduced the hyperoxia-induced increase in NF-κB expression. Data are shown as the mean ± SD. ***p<.001. NS: normal saline; RA: room air.

The hyperoxia-reared rats treated with NS exhibited more NF-κB immunohistochemistry intensity than did the RA-reared rats treated with NS or cathelicidin (Figure 3(A)). Cathelicidin treatment reduced NF-κB immunohistochemistry intensity in the hyperoxia-reared rats. The semiquantitative analysis of NF-κB immunoreactivity in the kidney tissue revealed that the expression was significantly higher in the hyperoxia-treated rats than in the RA-treated rats (Figure 3(B)). Hyperoxia-induced NF-κB immunohistochemistry intensity was decreased after cathelicidin administrated.

### M1/M2 phenotype

**Figure 4.**
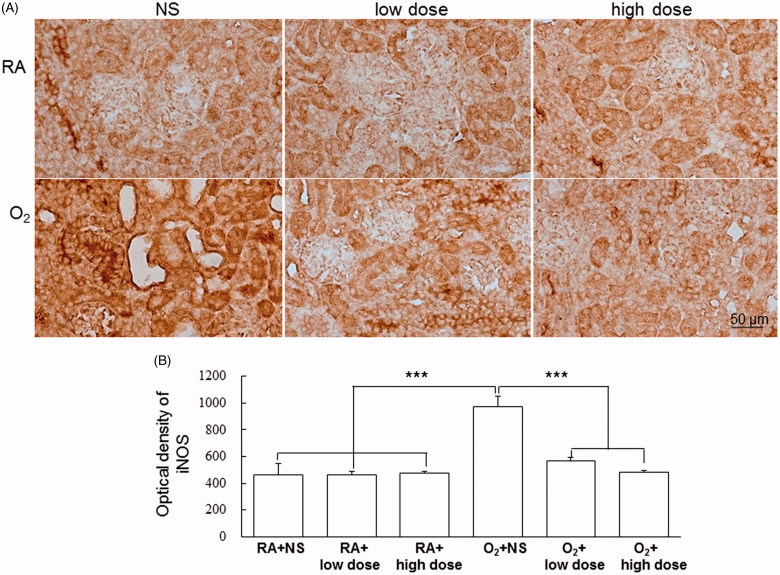
(A) Representative immunohistochemistry and (B) optical density of iNOS in the RA-reared and hyperoxia-reared rats and treated with NS or cathelicidin on postnatal day 7. The hyperoxia-reared rats treated with NS exhibited a significantly higher iNOS (M1 macrophage marker) density than did the RA-reared rats treated with NS or cathelicidin, and cathelicidin treatment reduced the hyperoxia-induced increase in iNOS optical density. Data are shown as the mean ± SD. ***p<.001. NS: normal saline; RA: room air.

**Figure 5.**
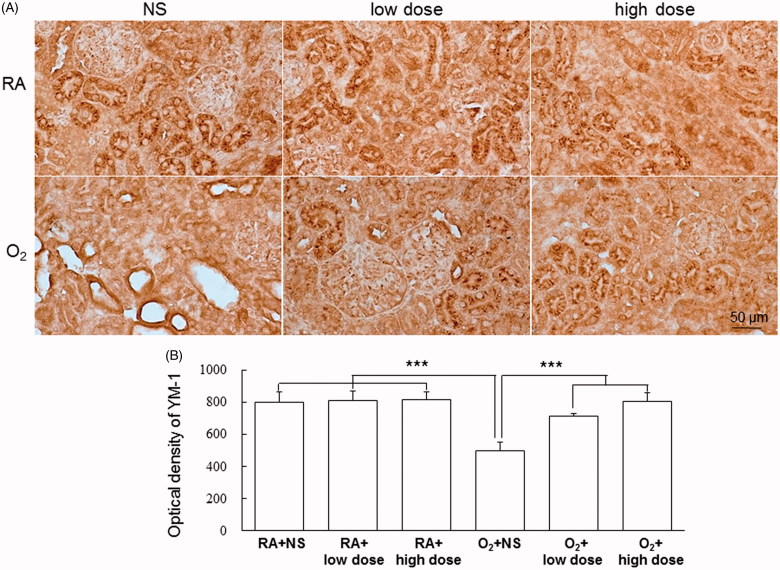
(A) Representative immunohistochemistry and (B) optical density of YM-1 in the RA-reared and hyperoxia-reared rats and treated with NS or cathelicidin on postnatal day 7. The hyperoxia-reared rats treated with NS exhibited a significantly lower Ym-1 density (M2 macrophage marker) than did the RA-reared rats treated with NS or cathelicidin, and cathelicidin treatment increased the hyperoxia-induced decrease in YM-1 optical density. Data are shown as the mean ± SD. ***p<.001. NS: normal saline; RA: room air.

Representative kidney sections stained with iNOS and Ym1 from the RA-reared and hyperoxia-reared rats and treated with NS or cathelicidin on postnatal day 7 are shown in Figures 4 and 5, respectively. The hyperoxia-reared rats treated with NS exhibited a significantly higher iNOS density (M1 macrophage marker) and lower Ym-1 density (M2 macrophage marker) than did the RA-reared rats treated with NS or cathelicidin, and cathelicidin treatment reversed the hyperoxia-induced M1/M2 macrophage polarization to RA levels.

### Collagen

**Figure 6.**
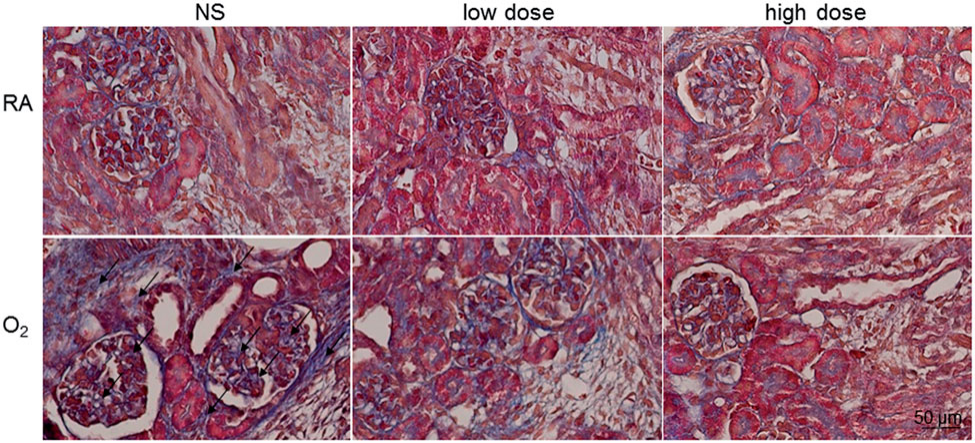
Masson’s trichrome staining in the RA-reared and hyperoxia-reared rats and treated with NS or cathelicidin on postnatal day 7. The hyperoxia-reared rats treated with NS exhibited increased collagen deposition in the glomerular mesangial matrix and tubular interstitium (arrow) of the kidneys and cathelicidin treatment reduced the hyperoxia-induced increase in collagen deposition. The color blue represents collagen fibers.

Masson’s trichrome staining verified the presence of kidney fibrosis in hyperoxia-exposed groups. The hyperoxia-reared rats treated with NS exhibited increased collagen deposition in the glomerular mesangial matrix and tubular interstitium of the kidneys and cathelicidin treatment reduced the hyperoxia-induced increase in collagen deposition (Figure 6).

## Discussion

Our previous study demonstrated that hyperoxia exposure during the first 7 d after birth induced tubular injuries and increased oxidative stress and the total collagen content in rat kidneys [4]. In this study, we treated newborn rats with cathelicidin, an antimicrobial peptide, and found that cathelicidin treatment attenuated kidney injury, which was evidenced by lower tubular injury scores and lower oxidative stress markers and collagen deposition in the cathelicidin-treated rats than in the NS-treated rats. The alleviation of kidney injury was accompanied by a reduction in kidney NF-κB expression. The major finding is that cathelicidin may protect against hyperoxia-induced kidney injury through the inhibition of the oxidative stress and NF-κB activity, thereby suggesting that cathelicidin may be a promising treatment modality against hyperoxia-induced kidney injury.

The mammalian kidney develops in three successive phases after embryogenesis: the pronephros, mesonephros, and metanephros phases. Nephrogenesis is complete by the 36th week of gestation in humans. In rodents, which have short gestation periods, the nephrogenesis is incomplete at birth and during the first postnatal 10 d [24]. Many preterm infants need high concentration of oxygen to treat respiratory distress at birth. Thus, newborn rats offer a useful model for studies of kidney development. In this study, we found rats reared in hyperoxia and treated with NS or cathelicidin exhibited significantly lower body weights on postnatal day 7 than did the RA-reared rats treated with NS or cathelicidin. The effects of cathelicidin administration on body weights have not been reported. Our study found that rats reared in hyperoxia exhibited respiratory distress, which may inhibit sucking mother's milk and reduced body weights.

Oxidative stress results from imbalance between reactive oxygen species production and antioxidant defenses. Hyperoxia increases the production of reactive oxygen species and subsequent oxidative stress activates NF-κB and initiates an inflammatory cellular response [25]. Oxidative stress causes DNA damage and lipid peroxidation which results in the generation of 8-OHdG [26]. In this study, we used immunohistochemistry to detect the renal oxidative stress marker 8-OHdG as its levels in target tissues are correlated with other oxidative stress markers [27]. We observed that hyperoxia significantly increased oxidative stress and NF-κB expression among rats reared in O_2_-enriched air compared with rats reared in ambient air. Cathelicidin treatment significantly reduced the hyperoxia-induced kidney injury and associated increase in oxidative stress and NF-κB. These results and our previous study indicate that hyperoxia-induced oxidative stress was one of the causes of kidney injury in this study [28].

Macrophages phenotype activation regulates the evolvement and prognosis of renal injury [29]. Activated M1 macrophages have pro-inflammatory effects and lead to tissue injury, while activated M2 macrophages have anti-inflammation and promotion of tissue repair [30]. However, hyperoxia effects on macrophage phenotypes in kidneys were not known. Given the link between oxidative stress and inflammation [31] and the number of macrophages correlates with renal disease progression [32], we used immunohistochemistry to detect the macrophage infiltration on the kidney tissue sections. Macrophage phenotype was assessed through immunostaining for iNOS (M1 macrophage marker) and Ym1 (M2 macrophage marker). The rats reared in hyperoxia and treated with in NS significantly exhibited more M1 macrophages compared with the rats reared in RA and treated with NS or cathelicidin. These results indicate that hyperoxia promotes the M1 phenotype in macrophages and suggest that the M1/M2 polarization mediates the hyperoxia effects in the developing kidney. Our study increases the understanding of the role of macrophage polarization in hyperoxia-induced injury to the developing kidneys.

In conclusion, this study demonstrated the therapeutic potential of cathelicidin in protecting against hyperoxia-induced kidney injury. This study also revealed that cathelicidin does not exert adverse effects on normal neonatal kidney development. Currently, no effective therapy is clinically available to prevent hyperoxia-induced kidney injury. Cathelicidin therapy may provide a novel strategy for preventing hyperoxia-induced kidney injury.

## Data Availability

The analyzed datasets generated during the study are available from the corresponding author on reasonable request.
